# Short-term hemodynamic effects of nebivolol in acute decompensated heart failure: a randomized clinical trial

**DOI:** 10.1186/cc10795

**Published:** 2012-03-20

**Authors:** R Puig, M Ochiai, J Cardoso, K Vieira, E Brancalhao, M Lima, A Pereira Barretto

**Affiliations:** 1 Hospital Auxiliar de Cotoxo - InCor - HCFMUSP, São Paulo, Brazil

## Introduction

Acute decompensation heart failure in patients in use of β-blocker has become frequent and maintenance of this drug remains controversial, mainly in low cardiac output. Nitric-oxide-dependent vasodilation of nebivolol could be useful in this situation.

## Methods

We evaluated hospitalized patients with acute decompensated heart failure, NYHA IV, EF <0.45, in use of dobutamine and carvedilol. Intervention: patients were randomly assigned to carvedilol maintenance or exchange to nebivolol according to Table 1. Hemodynamic parameters were compared using a noninvasive model flow technique (Nexfin^®^; BMEYE), 24 hours before, 6 and 24 hours after the randomization. *P *< 0.05 was significant.

**Table 1:** 

Carvedilol	Nebivolol
6.25 mg/bid	2.5 mg/qd
12.5 mg/bid	5.0 mg/qd
25.0 mg/bid	10.0 mg/qd

## Results

**Figure 1:**
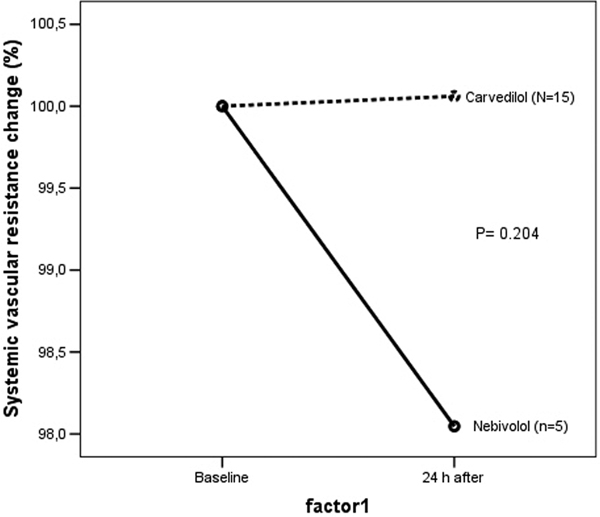
**Systemic vascular resistance change: nebivolol high×nebivolol low or carvedilol**.

We selected 30 patients, 75% men, age 56.0 (SD = 13.0) years, ejection fraction 23.4 (SD = 7.2)%, ischemic myocardiopathy present in 16.7%, Chagas disease in 40% and 43.3% of patients were nonischemic/non-Chagas. Baseline indexed systemic vascular resistance was 2,255.9 (SD = 792.4) dynes.second/cm^5^/m^2^, and cardiac index was 2.7 (SD = 0.6) l/minute/m. In the nebivolol group (*n *= 15) the indexed systemic vascular resistance reduced 0.6% and in the carvedilol group (*n *= 15) it reduced 5.0% in 24 hours (mean difference 4.4%; 95% CI: -12.6 to 21.4%; *P *= 0.513). The cardiac index maintained unchanged (*P *= 0.274). Comparing patients that received a high dose of nebivolol (5 to 10 mg/day) to those with a low dose (<5 mg/day) or carvedilol, we observed a tendency to superiority of high dose in reduction of systemic vascular resistance, although not statistically significant (Figure 1).

## Conclusion

Short-term nebivolol use in decompensated heart failure was hemodynamically safe. Further studies should be done to clarify this matter.

